# Ligand binding pocket of a novel Allatostatin receptor type C of stick insect, *Carausius morosus*

**DOI:** 10.1038/srep41266

**Published:** 2017-01-24

**Authors:** Burcin Duan Sahbaz, Osman Ugur Sezerman, Hamdi Torun, Necla Birgül Iyison

**Affiliations:** 1 Boğaziçi University Department of Molecular Biology and Genetics, Istanbul, 34342, Turkey; 2 Sabancı University Faculty of Engineering and Natural Sciences, Istanbul, 34956, Turkey; 3 Boğaziçi University Department of Electrical and Electronics Engineering, Istanbul, 34342, Turkey

## Abstract

Allatostatins (AST) are neuropeptides with variable function ranging from regulation of developmental processes to the feeding behavior in insects. They exert their effects by binding to cognate GPCRs, called Allatostatin receptors (AlstR), which emerge as promising targets for pesticide design. However, AlstRs are rarely studied. This study is the first reported structural study on AlstR-AST interaction. In this work, the first C type AlstR from the stick insect *Carausius morosus* (CamAlstR-C) was identified and its interaction with type C AST peptide was shown to be physically consistent with the experimental results. The proposed structure of CamAlstR-C revealed a conserved motif within the third extracellular loop, which, together with the N-terminus is essential for ligand binding. In this work, computational studies were combined with molecular and nano-scale approaches in order to introduce an unknown GPCR-ligand system. Consequently, the data obtained provided a reliable target region for future agonist/inverse agonist studies on AlstRs.

Allatostatin receptors (AlstR) are insect GPCRs, which function in inhibition of Juvenile Hormone (JH) synthesis in *corpora allata* during development. They also play a role during oogenesis, vitellogenesis and muscle contraction in midgut, either directly or indirectly via inhibiting JH^1^,^2^,^3^. It has been shown that oral administration of Allatostatin (AST) or its analogs reduce survival and nymph production in pea aphids^4^. Thus, the crucial role of AlstRs during key developmental and metabolic processes renders them potential targets for pesticide design. AlstRs identified so far have been grouped in three distinct classes (A, B and C types) according to their activating ligands. Type C AlstRs are homologous to mammalian somatostatin receptors (SSTR) which are known to inhibit growth hormone secretion in the hypothalamus^5^. Type A and B AlstRs are closely related to galanin and bombesin receptors, respectively. The physiological function of AlstR-AST systems varies according to species-specific, tissue-specific and developmental stage-specific expression. For instance, even though there are two AlstR-C paralogs in *D. melanogaster*, their cognate peptide AST-C does not inhibit JH in larval stage of the organism^6^. On the other hand, *Manduca sexta* AST-C peptides are shown to inhibit JH synthesis in different species and at developmental stages^7^. AlstRs belong to the rhodopsin-like family of GPCRs (Family A). Rhodopsin is the first GPCR for which the 3D structure was resolved by X-ray crystallography^8^ and therefore became a model for subsequent structural studies. Yet, X-ray and NMR approaches to reveal protein structure are very challenging for GPCRs due to their hydrophobic properties. Recent developments in bioinformatics have facilitated structural studies on GPCRs. For instance, Constanzi compared the crystal structure of the beta-2 adrenergic receptor with *in silico* models and proved its applicability to drug design^9^.

In order to support bioinformatics predictions and to identify the ligand binding pocket of GPCRs, random mutagenesis and subsequent activity assays are performed. In addition, molecular-physical approaches, such as Atomic Force Microscopy (AFM), enable quantitative analysis of the binding concept. However, working with purified GPCR molecules is not feasible because their dynamic properties may change when isolated from the lipid bilayer. Intermediate solutions such as integrating them into liposomes may provide a solution^10^,^11^. Yet, in order to understand their proper physiological responses GPCRs should be examined directly on the cell surface. Single molecule force spectroscopy via AFM has been used successfully on living cells to measure the strength of ligand binding of transporter proteins^12^ and integrins^13^ while the same studies on GPCRs are limited^14^.

This study presents a combined computational, molecular and nano-scale approach. A novel type C AlstR was identified from the Indian stick insect, *Carausius morosus*. The combination of bioinformatics, site directed mutagenesis and single molecule force spectroscopy (SMFS) allowed us to reveal its binding pocket. The methodology used in this study may provide an applicable method for further studies on the binding pocket of GPCRs, that would allow for the design of agonist/inverse agonists.

## Results

### Allatostatin receptor of *Carausius morosus*

**Figure 1:**
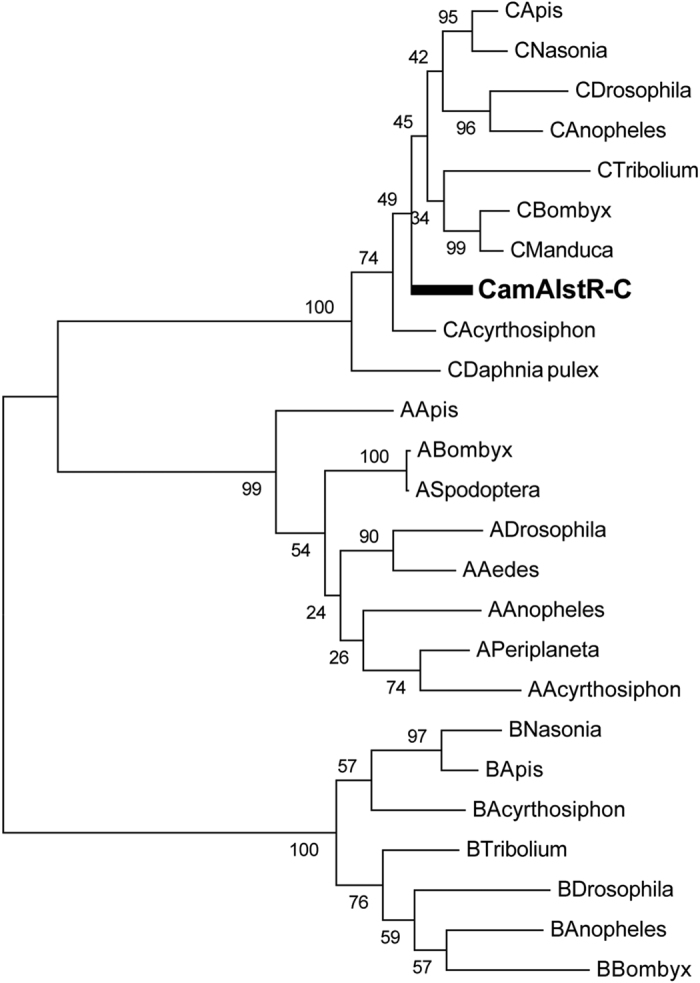
Phylogenetic analysis of AlstR identified in *Carausius morosus*. The evolutionary analysis of CamAlstR-C was inferred by using the Maximum Likelihood method in MEGA5. The letters (A,B and C) before the genus names correspond to the types of receptors. Ex; CDrosophila: AlstR type C of *Drosophila* genus. The branch in bold represents type C AlstR of *C. morosus.*

A novel GPCR mRNA was isolated from *C. morosus* (GenBank accession number: KX255655) and identified as a transcript of one of the AlstRs by phylogenetic analysis. The longest open reading frame (ORF) of this mRNA was 441 amino acids in length. Maximum likelihood analysis showed that this novel GPCR was closely related to type C AlstRs of other insect species (Fig. 1), which are homologous to mammalian SSTRs. Therefore, the receptor was named the type C Allatostatin Receptor of *Carausius morosus*, CamAlstR-C.

### Secondary and 3D structures of CamAlstR-C

**Figure 2:**
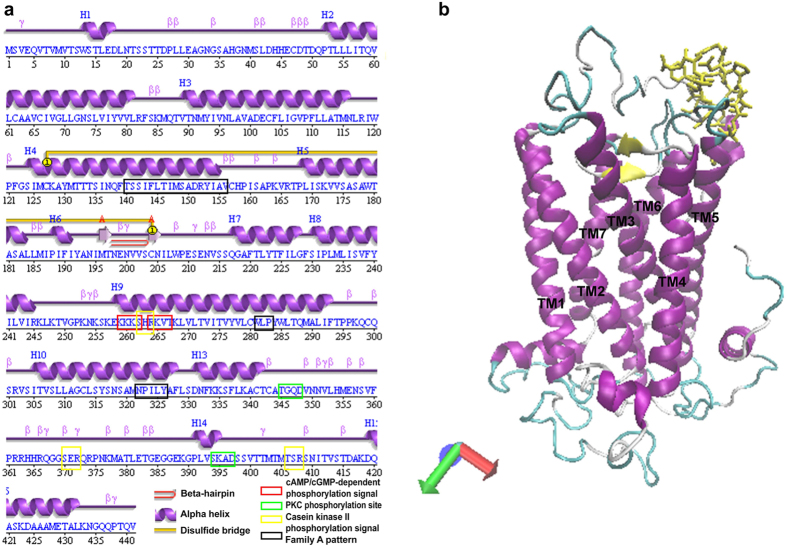
Structural elements of CamAlstR-C. (**a**) The sequence motifs that were detected in combination of PDBsum and MotifScan. The total protein sequence contains 15 helices (purple ribbons) constituting 7 transmembrane domains and one intracellular C-terminal helix. The receptor has a typical disulfide bridge (yellow connection line) between 127 C and 204 C. There exists a beta hairpin (red U turn) in ECL2 region. The other motifs are shown in rectangular frames. (**b**) 3D structure predicted in I-TASSER. TM helices are colored purple, beta-hairpin is yellow in new cartoon representation. The ligand is shown in yellow licorice structure. The green-grey loops represent the ICL and ECL regions.

Secondary structure prediction revealed that CamAlstR-C is composed of 15 helices, 13 of which constituted seven transmembrane (TM) domains (Fig. 2a). The presence of DRY motif on TM3, NPILY motif on TM7 and WLP motif on TM6 suggests that CamAlstR-C belongs to Family A GPCRs. A disulfide bridge between C127 of TM3 and C204 of second extracellular loop (ECL2) is another typical feature of this family (Supplementary Fig. S1). In addition, a beta-hairpin structure on ECL2 was present on CamAlstR-C structure. Additional structural features suggested the relationship to SSTRs. For instance, TM6 shows putative cAMP- and cGMP-dependent phosphorylation sequences (KKKS and RKVT) and a potential PKC phosphorylation sequence (SHR). At the C-terminal intracellular region, putative PKC phosphorylation signals (SER and TSR) and casein kinase II phosphorylation signals (TGQD and SKAD) were detected.

Even though the secondary structure predictions provided insights regarding the classification of this GPCR, the 3D structure was required to carry out functional analyses. There were no available structures of AlstRs in PDB. Therefore, homology modeling was performed to generate a 3D structure model of CamAlstR-C. The 3D structure model that showed the highest confidence and TM-scores is shown in Fig. 2b. However, the N-terminal region could not be modeled with high confidence since it shared low similarity with existing PDB structures (Supplementary Fig. S2) but could be predicted using *ab initio* calculations. Relevance of the model was assessed by calculating the root mean square Z-score for bond angles (1.526), bond length Z-score (0.997) and Ramachandran Z-score (−0.051) using the WhatIF server. The resulting values were similar to that of high resolution well refined structures.

### N-terminus, ECL2 and ECL3 were predicted to constitute the peptide binding pocket

**Figure 3:**
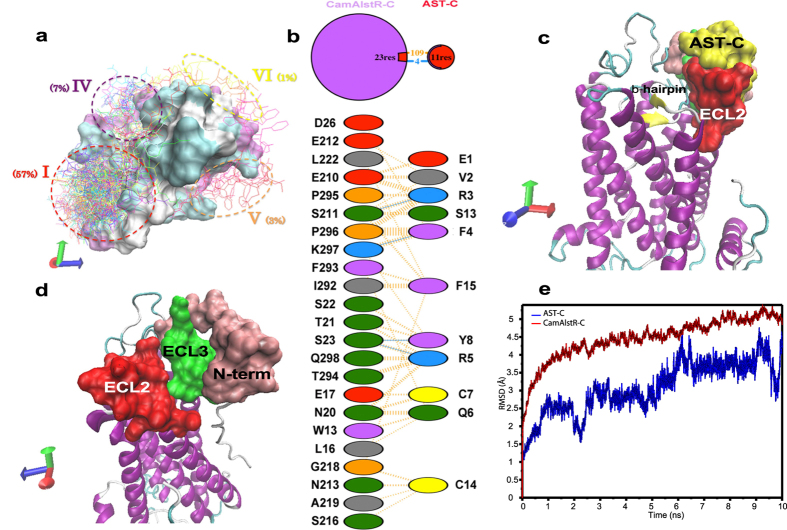
Constituents of putative binding pocket. (**a**) Clustering of different docking poses (n = 100) of AST-C on CamAlstR-C showed six different regions four of which were on the extracellular region. (**b**) Interacting residues of the most probable binding pocket were obtained from LIGPLOT. 3D structure of the binding pocket (**c**) with and (**d**) without the ligand was composed of ECL2 (red), ECL3 (green) and N-terminal loops (pink). (**e**) Molecular dynamic simulations for 10 ns for the ligand and the receptor showed that the RMSD reached a maximum of 4.5 and 5.5 Å, respectively.

AST-C peptide, the putative ligand of CamAlstR-C, was docked onto the receptor more than 100 times. The most probable docking region (I) contributed to 57% of the docking poses (Fig. 3a). The binding pocket of this pose was surrounded by the N-terminus, ECL2 and ECL3 residues (Fig. 3b and d). The beta-hairpin structure of CamAlstR-C formed an accessible binding pocket (Fig. 3c and d), unlike Rhodopsin, yet similar to C-X-C chemokine receptors (CXCRs). Molecular dynamics simulations were carried out for 10 ns to study the dynamics of the interaction between the ligand and the receptor. RMSD of the receptor was between 4 and 5.5 Å, and that of the ligand was between 1.5 and 4.5 Å showing a stable interaction between the two (Fig. 3e). However, when the ligand poses were taken from the second and third probable docking regions (II and III) RMSD values increased to 22 and 7.5 Å, respectively (Supplementary Fig. S3). The same simulations were performed to compare the interactions between the N-terminus deleted receptor and AST-C peptide and also between the wild type receptor and AST-A peptide. The resulting data were comparable with the results obtained from second and third region poses (Supplementary Fig. S3).

### Experimental support for AST-C interaction with CamAlstR-C

**Figure 4:**
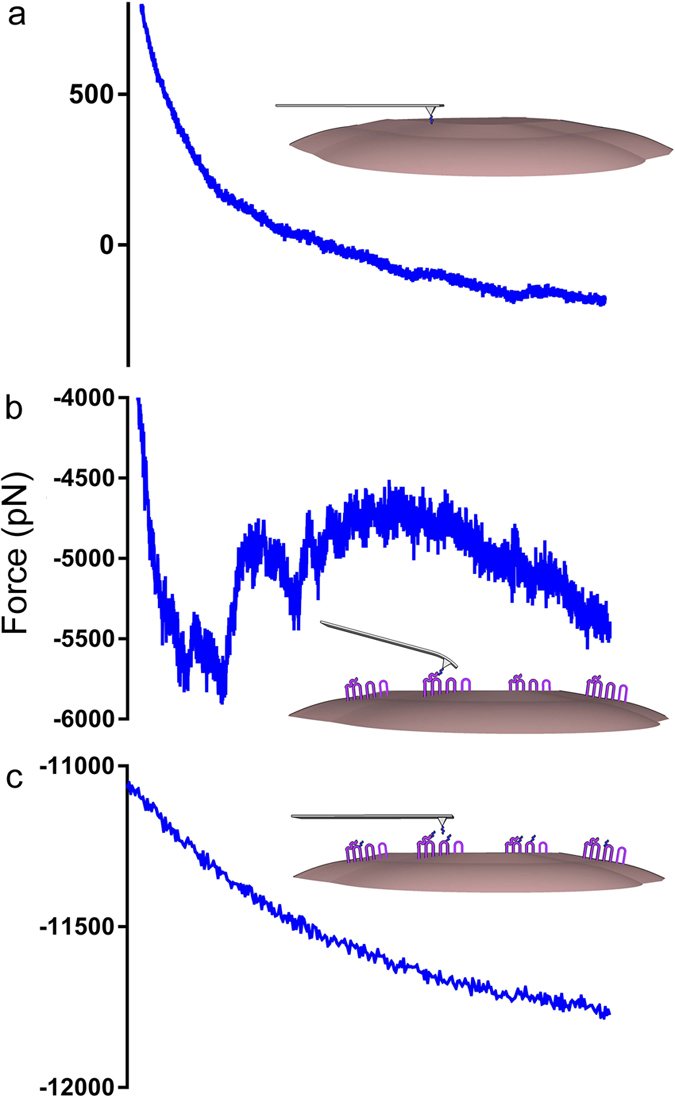
Specific interaction between AST-C and CamAlstR-C. Huh7 cells were transfected with empty plasmid (**a**) or CamAlstR-C construct (**b,c**). (**a**) Huh7 cells that were transfected with empty plasmid did not show unbinding events in AFM experiments. (**b**) CamAlstR-C expressing cells showed specific unbinding events with AST-C peptide. (**c**) After peptide wash of CamAlstR-C expressing cells, the unbinding events were lost again.

**Figure 5:**
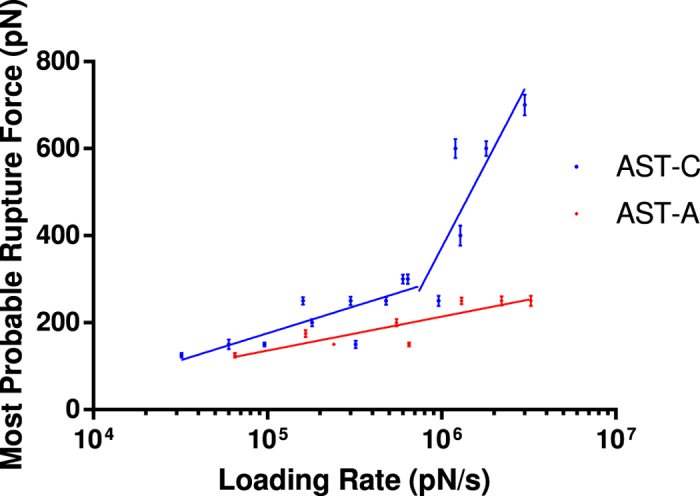
Comparison of interactions of AST-C and AST-A peptides with CamAlstR-C. Dynamic strength of interactions between CamAlstR-C and two peptides (AST-C, blue; AST-A, red) were plotted in AFM experiments. Most probable rupture forces were obtained in different loading rates. Error bars (s.e.m.) were calculated as the mean error of force histograms (n > 20).

**Figure 6:**
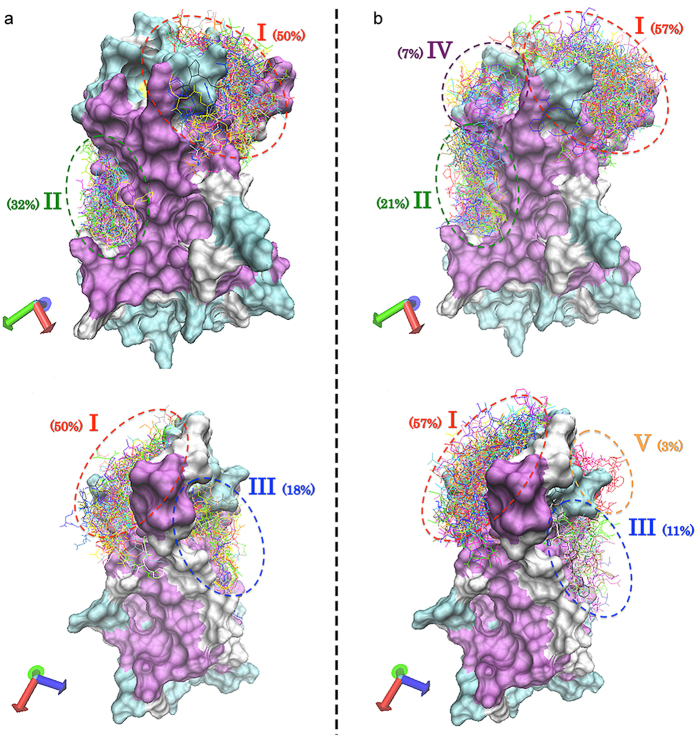
Docking poses of AST-C and AST-A on CamAlstR-C. (**a**) AST-A and (**b**) AST-C peptides (line representations) were docked onto CamAlstR-C (surface representation) and different poses (n = 100) were clustered into three and five distinct regions, respectively.

In order to propose a physical interaction between CamAsltR-C and AST-C, AFM-based force spectroscopy experiments were performed following *in silico* analyses. After expression of CamAlstR-C in mammalian Huh7 cells, immunofluorescence showed its localization to the cell surface, suitable for ligand binding experiments (Supplementary Fig. S4). AFM experiments performed on transiently CamAlstR-C expressing cells showed specific interaction between CamAlstR-C and AST-C peptide (Fig. 4). In the utilized AFM set up (Supplementary Fig. S5), the cells were attached to a solid surface, such as glass coverslips, and AST-C was bound to the cantilever. The AFM experiments were performed by actuating a cantilever functionalized with AST peptides repeatedly over the cells. The interaction of the peptides with the receptor resulted in deflection of the cantilever. The distance between the base of the cantilever and the cell surface was recorded together with the deflection of the cantilever during the experiments. The number of unbinding events on control cells transfected with empty plasmid (nearly 0 unbinding events in 100 retraction curves) was much lower than that on the cells transfected with CamAlstR-C (approximately 25 unbinding events in 100 retraction curves). An additional control was prepared by saturating the cells with excessive AST-C peptide. Under these conditions the resulting frequency of unbinding events was similar to that of the empty plasmid transfected control cells. In order to compare the specificity for AST-C peptide, commercially available AST-A was also used and showed lower unbinding forces with CamAlstR-C (Fig. 5). The same *in silico* analysis steps were performed for AST-A peptide and the binding region was found to be the same as that of AST-C (Fig. 6). In summary, CamAlstR-C and AST-C exhibited specific interaction *in vitro* and interaction of CamAlstR-C with AST-C was stronger than the interaction with AST-A.

### IXTPP motif in ECL3

**Figure 7:**
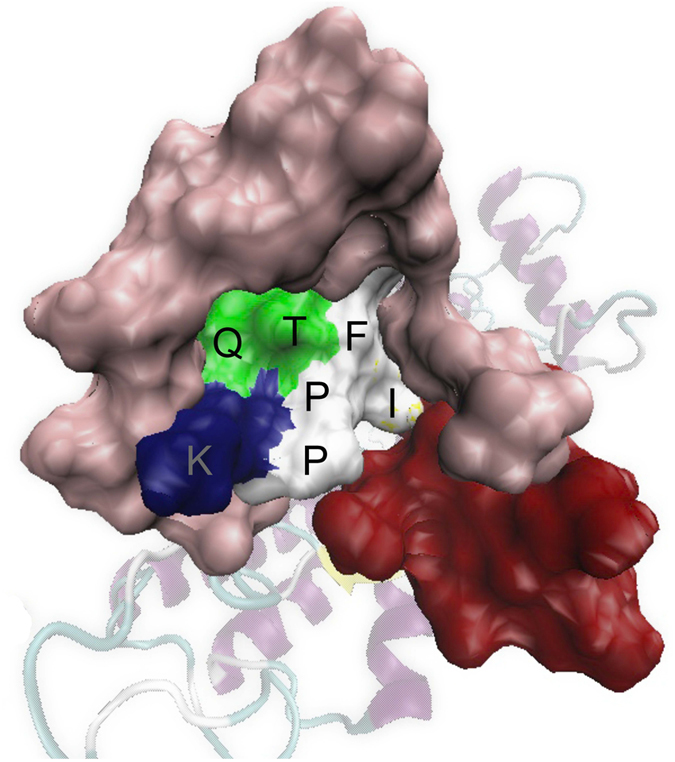
Location of ECL3 inside the binding pocket. The interacting residues on ECL3 (IFTPPKQ) were located in the middle of the binding pocket. The surface representation of binding pocket showed that the interacting portion of ECL3 was surrounded by N-terminal region (pink) and ECL2 (red). The amino acids were colored according to their properties (i.e. nonpolar residues white, polar ones green and basic residue as blue).

Our bioinformatics analysis revealed that the residues responsible for the interaction with the ligand were distributed around the N-terminus (WSTLEDLNTSSTTD), ECL2 (ESENVSSQGAFTL) and ECL3 (IFTPPKQ). Therefore, these regions were analyzed for evolutionary conservation with other insect species. Yet, the N-terminal regions were highly variable, but showed certain genus specific conservation (Supplementary Fig. S6). The predicted interacting residues on ECL2 were not conserved but located next to a highly conserved region (Supplementary Fig. S7). However, the residues on ECL3 showed high conservation and the structure model indicated that these residues were in the center of the proposed binding pocket (Fig. 7). Five amino acids of ECL3, IFTPP, were highly conserved like a motif IXTPP (Supplementary Figs S8 and S9).

### Binding force comparisons indicated the importance of IXTPP

**Figure 8:**
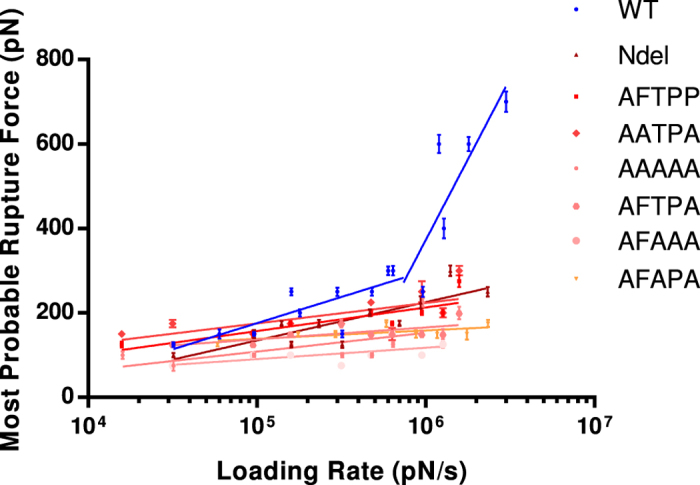
Force-time curves of mutant CamAlstR-C forms. Dynamic strength of interactions between AST-C with mutant forms were compared with that of the wild type receptor in AFM experiments. Blue data points and line represent the wild type receptor. The other data points in the legend were colored and ordered in increasing K_off_ order. Error bars (s.e.m.) were calculated as the mean error of force histograms (n > 20).

In order to verify *in silico* data, site-directed mutagenesis was performed on CamAlstR-C IXTPP motif. Different combinations of Alanine substitutions (AFTPP, AFTPA, AFAPA, AATPA, AFAAA, AAAAA) and a version in which the N-terminus was deleted (Ndel) were used for AFM measurements (abbreviations and descriptions of mutations are given in Supplementary Table S1). As predicted, all mutated receptor sequences exhibited lower unbinding forces (f_u_) than WT CamAlstR-C (Fig. 8). This was also true for Ndel form, which showed decreased f_u_ for AST-C. In order to compare off-rates (K_off_) and energy barrier widths (*x*_*β*_) of WT and mutant receptor forms, loading rates were adjusted from 16.000 to 3.000.000 pN/s for each experiment. The f_u_ of a specific interaction in AFM was varied according to the loading rate applied. However, if the strength of interaction decreased, the change in f_u_ also decreased and in a way that was undetectable from unspecific interactions. K_off_ and *x*_*β*_ values were calculated with the equations obtained from curves in Figs 5 and 8, via formulas (3 and 4). As a result, wild type CamAlstR-C with AST-C peptide showed two different equations in different loading rates. In smaller loading rates its K_off_ and *x*_*β*_ were calculated as 2,00E + 10 s^−1^ and 0.828 Å, respectively. In higher loading rates, these values were 3,33E + 9 s^−1^ and 0.138 Å, respectively. These two equations indicated that WT CamAlstR-C and AST-C interaction had a two-step energy barrier in unbinding. However, this two-step process disappeared in the mutant receptor interaction. In addition, Bell’s parameters for each mutant receptor showed that the strength of interaction decreased in all of the mutant receptor forms. K_off_ and *x*_*β*_ parameters for Ndel, AAAAA, AFAAA, AATPA, AFAPA, AFTPA and AFTPP were given in Supplementary Table S2.

## Discussion

Phylogenetic analysis of the isolated *C. morosus* sequence revealed that it was more closely related to type C AlstRs than to type A and B. Type A and C AlstRs were closer to each other than to type B. Moreover, type B AlstRs have diverged before A and C type differentiation. In this study, a novel GPCR from *C. morosus* was identified and the phylogenetic analysis revealed that it belonged to type C AlstRs. To study the sequence-structure-function relationship of this GPCR, several bioinformatics analyses were conducted.

Multiple alignment studies on the N-terminus of AlstR-C resulted in a notable conservation. A genus-specific conservation pattern was detected within 3 genera, and CamAlstR-C showed a significant diversity from this profile. Previous studies on AST ligands had shown that *Manduca sexta* AST-C could cross-activate type C receptors in different species and at developmental stages^7^,^15^. AFM studies on N-del CamAlstR-C have revealed that the N-terminal region was important for ligand binding. In our bioinformatics docking analysis, AST-C showed a tendency to drift apart from the proposed binding pocket region when its N-terminus was deleted (data not shown). 3D structure predictions suggest that the first part of the N-terminal region is in close proximity to ECL2 and ECL3, and may play a critical role in the interaction with the ligand. We suggest that the N-terminal sequence of CamAlstR-C might not define ligand-specific activity per se, but may support proper 3D conformation and rigidity of the binding pocket that leads to efficient ligand binding.

CamAlstR-C shows typical features of the Rhodopsin-like family of GPCRs. Palczewski’s Rhodopsin exhibits a beta-hairpin turn on ECL2^8^, forming a closed lid on the ligand binding region. In the same family, studies on β1 and β2-adrenergic receptors have demonstrated the presence of a helix on ECL2 that forms a disulfide bridge with the extracellular end of TM3^16^. This disulfide bridge appeared to be conserved within the family and was also predicted for CamAlstR-C. However, in CamAlstR-C it seemed to form a pocket-like vacancy on the extracellular region, more similar to CXCR4. In CXCR4 the same beta-hairpin loop as that of rhodopsin, but the disulfide bond between ECL2 and the N-terminus defined an open entrance for the ligand^17^. The accessible, rather than capped binding pocket of CamAlstR-C may be responsible for the reversible and temporary binding with the ligand^18^,^19^.

Ligand interacting residues (from 210 to 222) on ECL2 were less conserved. Highly conserved residues on ECL2 were N205, I206, W208 and P209 (NIXWP), but did not interact with the ligand in our docking analysis. Thus, this conserved NIXWP motif may have an important role in conformational exposition of ligand interacting residues.

CamAlstR-C showed some functional motifs in accordance with mammalian SSTRs. For example; in the intracellular regions of CamAlstR-C cAMP and cGMP dependent phosphorylation, PKC phosphorylation and Casein kinase II phosphorylation signals could be detected. These motifs provide clues for future studies on downstream signaling pathways. SSTRs are known to couple with pertussis toxin-sensitive G proteins which inhibit adenylate cyclase activity and lead to reduced levels of cAMP in the cell^20^. As Kumar and Grant^21^ reviewed for the motifs, SSTRs can also activate PKC-dependent and Casein kinase II-dependent pathways. However, the functionality and signaling pathway of CamAlstR-C await further analysis.

The slopes of curves obtained from AFM dynamic force spectra allow for a direct prediction of the affinity of CamAlstR-C for a ligand. The increase in the unbinding forces (f_u_) with the increase in loading rates showed a specific interaction between two molecules. If there is no change in f_u_ while loading rates are increasing, this means that the interaction is non-specific^22^. AFM experiments performed with the WT CamAlstR-C and AST-C peptide showed that there was specific interaction between these two molecules. In addition, this specific interaction was also present with the AST-A peptide, which typically activates type AlstRs. However, the latter interaction was weaker as the f_u_ decreased for the same loading rates. In higher loading rates (>10^6^ pN/sec), AST-C unbinding forces showed a sharp increase, changing the equation of the curve. This may be an indication of two-step energy barrier in this unbinding event as reviewed by Evans, Williams and Lee^23^,^24^. The complexity of this unbinding may stem from i) breakage of the disulfide bond of AST-C, ii) conformational change on CamAlstR-C or iii) flexibility of the binding pocket of CamAlstR-C.

Mutant receptors showed weaker interaction with AST-C peptide than the wild type receptor. AFTPA, AFAAA and AFAPA mutations showed similarity in their K_off_ values (1,00E + 11, 1,00E + 11 and 1,11E + 11 s^−1^ respectively) and had higher K_off_ values than AFTPP, AATPA and AAAAA mutations (5,00E + 10 s^−1^ each). Consequently, the residue positions could not be ranked according to their importance, and it was preferred to mention the importance of this five-residue region as a whole. Further investigations can be performed to reveal whether these residues behave as a motif or can be equally important for the ligand interaction. In all mutations, the two-step unbinding property disappeared. In Ndel CamAlstR-C 50 amino acids were deleted. The results showed a higher K_off_ value (2,50E + 10 s^−1^) than that for WT receptor, but lower than that for substitution mutations. Similar to previously reported studies on the importance of the N-terminus in ligand binding of Rhodopsin-like GPCRs^25^,^26^, it was concluded that the N-terminus of CamAlstR-C is also important for the ligand binding but not as vital as the IXTPP motif on ECL3.

Interaction of two purified molecules is studied in many ways. However, GPCRs are not easy to purify and have too many dynamic conformations. Studying them directly on cells gives more realistic results due to their natural environment. Composition of lipid bilayers is cell-dependent and defines functionality of membrane proteins. Therefore, it is mostly recommended to study GPCRs on a real lipid environment where they are still functional^27^.

AFM provides a simple method to study GPCR-ligand interaction under physiological conditions. Single Cell Force Spectroscopy uses single cells attached on the cantilever and measures adhesive forces^28^. However, some cells, such as adherent cancer cells, require the presence of adjacent cells to feel in an appropriate environment and this situation defines their adherence, stability and viability in experimental conditions. Cancer cell lines in general are strongly adhesive, stable, viable and easily transfectable. However, they need a confluent environment to provide these properties. Huh7 cells, which are hepatocellular carcinoma cells, provided these properties for the duration of the AFM experiments.

However, living cells may also influence critical parameters, for instance the flexibility of membrane might affect force-distance (FD) curves^29^. Despite these unanswered questions, good evidence for interaction between CamAlstR-C and AST-C on the surface of a live cell could be obtained. The pattern of FD curve of retraction step is defined by the elastic properties of the cells and interpretation of these curves for Huh7 cell lines should be studied further.

While working on living cells, other limitations such as high expression and correct orientation of the receptor on the cell membrane should be considered. These factors are important for proper binding with the ligand. In order to confirm its expression and orientation, immunocytochemistry or immunofluorescence can be performed or a fluorescent microscope can be combined with AFM set up^30^. It was verified that CamAlstR-C has been transported to the membrane of Huh7 cells via immunofluorescence. In addition, specific unbinding events obtained in preliminary AFM experiments with control groups revealed that the orientation of CamAlstR-C should be appropriate for ligand binding.

In summary, a novel type C AlstR from *C. morosus* was isolated and its predicted structure and binding pocket for AST-C peptide were characterized. This study utilized the advances in structure-based studies and single-molecule based approaches in order to obtain detailed information about the AlstR-AST interaction. In addition, for the first time, an applicable method for agonist/antagonist binding studies on a novel GPCR in their preferred physiological condition was provided.

## Methods

### Identification of TM of CamAlstR-C

Adult specimens of *C. morosus* were euthanized in liquid nitrogen and were stored at −80 °C until processing. Total RNA was isolated with RNeasy Mini Kit (QIAGEN, Redwood City, CA, USA) according to the manufacturer’s instructions. cDNA synthesis was performed with ImProm-II™ Reverse Transcription System using oligo dT primers (Promega, Medison, Wis, USA) according to the manufacturer’s instructions. 5 μl of cDNA was used for amplification of the TM region using degenerate primers 5′-GCGGAATTC(C/T)(T/A)(C/T)TGGCCXTT(C/T)GG-3′ and 5′-GACGGATCC(G/A)AAXGGXA(G/A/T)CCA(G/A)CA-3′^31^ and Phusion High-Fidelity DNA Polymerase (New England Biolabs, MA, USA). PCR products of the expected lengths (500–700 bp) were cloned into pGEM-T Easy vector system (Promega, Medison, Wis, USA) and sequenced.

### cDNA synthesis and RACE

In order to identify 3′ and 5′ ends of AlstR mRNA, Rapid Amplification of cDNA Ends (RACE) was performed. The PCR reactions were performed by 5′/3′ RACE Kit 2nd Generation (Roche, Basel, Switzerland) according to the manufacturer’s instructions. For 5′ RACE specific primers were SP1 5′-AAGCGGAGCACCACGTAGAT-3′ and SP2 5′-GACGAGGCCGTTGCCGAGCAG-3′. For 3′ RACE specific primers were SP4 5′-CTGCACATGGAGAACAGCGTG-3′ and SP5 5′-TTCCCGCGGCGCCACCCCGG-3′.

### Amplification of the open reading frame

The fragment sequences from 3′ and 5′ RACE reactions and sequence of TM region were put in order and the longest open reading frame was predicted in a six-frame translation web server (Bioline). New primers were designed to include the first ATG codon (5′-AAACTAGTCAAAATGTCTGTGGAACAAGTGACG-3′) and the Stop codon (5′-TTTGAATTCTTGGATCCTCTACACCTGGGTCGGCTG-3′) to amplify the longest ORF using Phusion High-Fidelity DNA Polymerase (New England Biolabs, MA, USA). The amplified product was cloned into a previously modified pcDNA3/HA vector. For immunofluorescence (IF) experiments, the same insert was cloned into an SYFP containing pcDNA3 vector.

### Site directed mutagenesis and deletion of the N-terminus

In order to replace the amino acids between residues 292 and 296 with Alanines, site-directed mutagenesis was performed using Phusion High-Fidelity DNA Polymerase (New England Biolabs, MA, USA) (primer list is given in Supplementary Table S3). PCR products were cloned into the pcDNA3/HA vector. On the 292–296 region, all amino acids (IFTPP) were substituted with Alanine residues in various combinations resulting in the series AFTPP, AFTPA, AATPA, AFAPA, AFAAA and AAAAA. For instance, AFTPA mutation code means that 292I and 296 P were only replaced with Alanine amino acids.

To delete the N-terminal 52 amino acids of the receptor, the ORF was amplified with a primer having an artificial ATG codon immediately upstream of the sequence complementary to the 52nd amino acid codon of the receptor (5′-AAAAAGCTTATGGACACAGACCAGCCGACG-3′) and the original Stop codon primer. This mutation was called Ndel.

### Cell lines and expression of CamAlstR-C

For IF and AFM experiments, CamAlstR-C was ectopically expressed in a human hepatocellular carcinoma cell line, Huh7. For both AFM and IF experiments these cells were grown on 18 mm coverslips. Transient transfections were performed with FuGENE HD Transfection Reagent (Promega, Medison, Wis, USA) with a 3:1 reagent:DNA ratio. Each transfection was controlled for efficiency with an EGFP-containing plasmid (pEGFP-N2) and for transfection, toxicity with an empty plasmid (modified pcDNA3/HA).

### Immunofluorescence

The CamAlstR-C was fused with SYFP on its C terminus and expressed in Huh7 cells. The cells were fixed in 4% paraformaldehyde (PFA) on ice for 15 min. Blocking was performed in 2% bovine serum albumin (BSA) for 1 hr at room temperature (RT). In order to mark the cell membrane α-ZO1 (Invitrogen, catalog number: 40–2200) was used in a dilution of 1:200 in 2% BSA. The secondary antibody was Alexa 555-conjugated α-rabbit IgG and used in 1:200 dilution in 2% BSA. Nuclei were stained with TO-PRO^®^-3 (Thermo Scientific) according to the manufacturer’s instructions. The samples were imaged and analyzed on a LEICA-SP5 confocal microscope (TCS, Leica, Germany).

### Atomic Force Microscopy

Atomic force microscopy experiments were performed in BioAFM Laboratory of Hamdi Torun in Electrical and Electronical Engineering Department of Boğaziçi University. PNP TR-20 (k = 80 pN/nm, NanoWorld) and OTR4 silicon nitride cantilever (k = 80 pN/nm, Bruker) were used. The cantilevers were incubated with 100 ng/ml AST-C peptide (98.45% purity) for 20 min at RT and washed with 1X PBS. For AST-A (>97% purity) binding experiments, OTR4 silicon nitride cantilever (k = 80 pN/nm, Bruker) was incubated in 1 mg/ml Allatostatin IV peptide for 15 min at RT. The distance between the cantilever tip and sample was adjusted to 1 mm via focusing of a single cell. On each sample, more than 50 recordings were counted. For each sample, data were collected on different loading rates (0.1 μm/s, 0.5 μm/s, 1 μm/s, 3 μm/s, 5 μm/s, 10 μm/s, 15 μm/s, 20 μm/s, 30 μm/s and 40 μm/s). WT and mutant receptor recordings (AFTPP, AFTPA, AFAPA, AATPA, AFAAA, AAAAA and Ndel) were performed in biological duplicates. AST-A peptide binding was performed for only the WT receptor. The experiment was calibrated in an empty area with no cells. The control groups were set as a sample with no CamAlstR-C expression (empty pcDNA3 transfected cells), a sample in which the cantilever penetrated the cell membrane, and another sample washed with excess peptide to saturate CamAlstR-C receptors. The unbinding events were evaluated against non-adhesive events and these were taken for force calculations. Force-distance (FD) curves were produced from potential-distance curves, according to the following formulas:


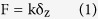


Here, k is the spring constant of the cantilever and δ_z_ is the cantilever deflection change in z axis. This deflection was calculated via the following formula from the calibration curve:


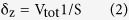


Here, S is optical lever sensitivity and obtained from the slope of the linear part of calibration curve on a stiff surface which is assumed to be “indefinitely hard”. V_tot_ is the potential difference when the cantilever moves one step towards and against the sample^24^. Histograms were generated for detection of the most probable rupture forces. These rupture forces were plotted against loading rates in dynamic force spectrum. Dissociation rate constants (K_off_) and (*x*_*β*_) were calculated via the formulas below:


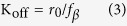



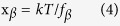


Here, *r*_*0*_ is the loading rate (pN/s) at zero force, *f*_*β*_ is the slope of the curve of dynamic force spectrum and *kT* is the Boltzmann constant (4.11 pN/nm).

### Phylogenetic analysis

Protein sequences of AlstRs from different insect species (Apis and Nasonia from Hymenoptera, Aedes, Anopheles and Drosophila from Diptera, Tribolium from Coleoptera, Bombyx, Spodoptera and Manduca from Lepidoptera, Acyrthosiphon from Hemiptera, Daphnia from Crustaceans, and Periplaneta from Dictyoptera) were taken for phylogenetic analysis. The ones that contained only partial protein sequences were excluded and only one receptor sequence was taken into account for each genus. Sequences from 8 Allatostatin A receptors (AlstR-A) from 8 species, 7 Allatostatin B receptors (AlstR-B) from 7 species and 9 Allatostatin C receptors (AlstR-C) from 9 species were aligned with CamAlstR-C and checked for the maximum coverage of similarity. The most conserved regions were included and the variable regions (mostly N-terminus and C-terminus) were deleted. Each type of AlstR was aligned and type-specific profiles were taken into profile-to-profile alignment via PAM matrix in ClustalWProf. CamAlstR-C was aligned with the final profile of 24 proteins, in sequencetoprofile via ClustalWProf and the alignment was exported. The evolutionary relatedness was inferred by using Maximum Likelihood method based on the JTT matrix-based model with 500 bootstrap replicas, in MEGA5. When the number of common sites was <100 or less than one fourth of the total number of sites, the maximum parsimony method was used; otherwise BIONJ method with MCL distance matrix was used. The tree was drawn to scale, with branch lengths measured in the number of substitutions per site.

### Binding motif discovery

Estimated constituents of the binding pocket, the N-terminal region, ECL2 and ECL3, were analyzed for conservation within other species. The N-terminal region was taken as the first 54 amino acids. AlstR-C N-terminal regions were taken from only 3 genera containing 16 different species (11 *Drosophila*, 3 *Anopheles* and 2 *Apis* species). These sequences were aligned in ClustalW with BLOSUM62 matrix. The alignments were aligned in ClustalWProf (Gonnet Matrix) in order to compare inter-genera conservations. At the end CamAlstR-C N-terminus was aligned to this profile via ClustalWProf.

### Secondary and 3D structure predictions

Transmembrane regions were predicted in TMHMM server v.2.0, PSIPRED and PDBSUM, and compared. Secondary structure motifs and functional motifs were obtained in ScanProsite (ExPASy) and Motif Scan (MyHits, SIB, Switzerland) respectively.

3D structure prediction of CamAlstR-C was performed on I-TASSER server^32^ in the default conditions. Other servers such as Robetta, Swiss Model, ModWeb and (PS)^2^ were also used to construct a 3D model and their predictions were compared with I-TASSER model. The ligand was also modeled in I-TASSER but with a constraint. This modeling was restrained for a distance of 2.05 Å between sulfur atoms of 7Cys and 14 C.

### Molecular docking

Docking analysis was performed in AutoDock Vina version 1.1.2^33^. The center of the grid box was chosen close to the extracellular part of the receptor and its dimensions were adjusted to cover all of the extracellular loops. Exhaustiveness of docking analysis was set for 8. The refined output was taken into LIGPLOT to obtain the possible interactions between the two proteins. Each docking was performed at least 100 times in order to cluster the ligand-approaching regions on the receptor.

### Molecular dynamics

CamAlstR-C pdb coordinates were loaded in Visual Molecular Dynamics (VMD) software^34^ with both AST-A and AST-C peptide coordinates. Ionization and solvation were performed and trajectory files were prepared. These trajectories were used in NAMD^35^. The simulations were run for 10 ns with 2000 minimization steps at 298 K.

## Additional Information

**How to cite this article**: Sahbaz, B. D. *et al*. Ligand binding pocket of a novel Allatostatin receptor type C of stick insect, *Carausius morosus*. *Sci. Rep.* **7**, 41266; doi: 10.1038/srep41266 (2017).

**Publisher's note:** Springer Nature remains neutral with regard to jurisdictional claims in published maps and institutional affiliations.

## Supplementary Material

Supplementary Information

## References

[b1] Audsley N., Matthews H. J., Price N. R. & Weaver R. J. Allatoregulatory peptides in Lepidoptera, structures, distribution and functions. J. Insect Physiol. 54, 969–80 (2008). 18377924 10.1016/j.jinsphys.2008.01.012

[b2] Herman W. S. & Barker J. F. Effect of mating on Monarch butterfly oogenesis. Experientia 33, 688–689 (1977). 862820 10.1007/BF01946578

[b3] Martín D., Piulachs M. D. & Bellés X. Inhibition of vitellogenin production by allatostatin in the German cockroach. Mol. Cell. Endocrinol. 121, 191–196 (1996). 8892320 10.1016/0303-7207(96)03864-6

[b4] Down R. E., Matthews H. J. & Audsley N. Effects of Manduca sexta allatostatin and an analog on the pea aphid Acyrthosiphon pisum (Hemiptera: Aphididae) and degradation by enzymes from the aphid gut. Peptides 31, 489–97 (2010). 19560498 10.1016/j.peptides.2009.06.017

[b5] Brazeau . Hypothalamic polypeptide that inhibits the secretion of immunoreactive pituitary growth hormone. Sci. (New York, NY) 179, 77–79 (1973). 10.1126/science.179.4068.77 4682131

[b6] Stay B. & Tobe S. S. The role of allatostatins in juvenile hormone synthesis in insects and crustaceans. Annu. Rev. Entomol. 52, 277–99 (2007). 16968202 10.1146/annurev.ento.51.110104.151050

[b7] Kramer S. J. . Identification of an allatostatin from the tobacco hornworm Manduca sexta. Proc. Natl. Acad. Sci. USA 88, 9458–9462 (1991). 1946359 10.1073/pnas.88.21.9458 PMC52737

[b8] Palczewski K. . Crystal Structure of Rhodopsin: A G Protein-Coupled Receptor. Science (80-.). 289, 739–745 (2000). 10.1126/science.289.5480.739 10926528

[b9] Constanzi S. On the applicability of GPCR homology models to computer-aided drug discovery: a comparison between in silico and crystal structures of the β2 -adrenergic receptor. J Med Chem 51, 2907–2014 (2008). 18442228 10.1021/jm800044k PMC2443693

[b10] Pfreundschuh M. . Identifying and quantifying two ligand-binding sites while imaging native human membrane receptors by AFM. Nat. Commun. 6, 8857 (2015). 26561004 10.1038/ncomms9857 PMC4660198

[b11] Alsteens D. . Imaging G protein-coupled receptors while quantifying their ligand-binding free-energy landscape. Nat. Methods 1 (2015). 10.1038/nmeth.3479 PMC5087271 26167642

[b12] Wildling L. . Probing Binding Pocket of Serotonin Transporter by Single Molecular Force Spectroscopy on Living Cells*. 287, 105–113 (2012). 10.1074/jbc.M111.304873 PMC3249061 22033932

[b13] Yang H. . Interaction between single molecules of Mac-1 and ICAM-1 in living cells: An atomic force microscopy study. Exp. Cell Res. 313, 3497–3504 (2007). 17803991 10.1016/j.yexcr.2007.08.001

[b14] Zhang J. . Single molecular recognition force spectroscopy study of a luteinizing hormone-releasing hormone analogue as a carcinoma target drug. J. Phys. Chem. B 116, 13331–7 (2012). 23094688 10.1021/jp306882r

[b15] Audsley N. & Weaver R. J. Neuropeptides associated with the regulation of feeding in insects. Gen. Comp. Endocrinol. 162, 93–104 (2009). 18775723 10.1016/j.ygcen.2008.08.003

[b16] Warne T. . Structure of a beta1-adrenergic G-protein-coupled receptor. Nature 454, 486–492 (2008). 18594507 10.1038/nature07101 PMC2923055

[b17] Wu B. . Structures of the CXCR4 Chemokine GPCR with Small-Molecule and Cyclic Peptide Antagonists. Science (80-.). 330, 1066–1071 (2010). 10.1126/science.1194396 PMC3074590 20929726

[b18] Tan E. M. . Selective and Quickly Reversible Inactivation of Mammalian Neurons *In Vivo* Using the Drosophila Allatostatin Receptor. Neuron 51, 157–170 (2006). 16846851 10.1016/j.neuron.2006.06.018

[b19] Ikrar T., Shi Y., Velasquez T., Goulding M. & Xu X. Cell-type specific regulation of cortical excitability through the allatostatin receptor system. Front. Neural Circuits 6, 2 (2012). 22319474 10.3389/fncir.2012.00002 PMC3262160

[b20] Patel Y. C. Somatostatin and Its Receptor Family. Front. Neuroendocrinol. 20, 157–198 (1999). 10433861 10.1006/frne.1999.0183

[b21] Kumar U. & Grant M. Somatostatin and somatostatin receptors. Results Probl. Cell Differ. 50, 137–84 (2010). 19859675 10.1007/400_2009_29

[b22] Le D. T. L., Guérardel Y., Loubire P., Mercier-Bonin M. & Dague E. Measuring kinetic dissociation/association constants between Lactococcus lactis bacteria and mucins using living cell probes. Biophys. J. 101, 2843–2853 (2011). 22261074 10.1016/j.bpj.2011.10.034 PMC3297779

[b23] Evans E. & Williams P. In Physics of bio-molecules and cells. Physique des biomolécules et des cellules (eds. Flyvbjerg F., Jülicher F., Ormos P. & David F.) 145–204, doi: 10.1007/3-540-45701-1_4 (Springer Berlin Heidelberg, 2002).

[b24] Lee C. K., Wang Y. M., Huang L. S. & Lin S. Atomic force microscopy: Determination of unbinding force, off rate and energy barrier for protein-ligand interaction. Micron 38, 446–46 (2007). 17015017 10.1016/j.micron.2006.06.014

[b25] Gupta S. K., Pillarisetti K., Thomas R. A. & Aiyar N. Pharmacological evidence for complex and multiple site interaction of CXCR4 with SDF-1α: Implications for development of selective CXCR4 antagonists. Immunol. Lett. 78, 29–34 (2001). 11470148 10.1016/s0165-2478(01)00228-0

[b26] Cho D. I. . The N-terminal region of the dopamine D2 receptor, a rhodopsin-like GPCR, regulates correct integration into the plasma membrane and endocytic routes. Br. J. Pharmacol. 166, 659–75 (2012). 22117524 10.1111/j.1476-5381.2011.01787.x PMC3417496

[b27] Bippes C. A. & Muller D. J. High-resolution atomic force microscopy and spectroscopy of native membrane proteins. Reports Prog. Phys. 74, 86601–43 (2011).

[b28] Taubenberger A., Friedrichs J. & Müller D. J. In Life at the Nanoscale: Atomic Force Microscopy of Live Cells (ed. Dufrêne Y.) 209–224 (Pan Stanford Publishing, 2011).

[b29] Sun M. . Multiple Membrane Tethers Probed by Atomic Force Microscopy. Biophys. J. 89, 4320–4329 (2005). 16183875 10.1529/biophysj.104.058180 PMC1366996

[b30] Madl J. . A combined optical and atomic force microscope for live cell investigations. Ultramicroscopy 106, 645–51 (2006). 16677764 10.1016/j.ultramic.2005.12.020

[b31] Auerswald L., Birgül N., Gäde G., Kreienkamp H. J. & Richter D. Structural, functional, and evolutionary characterization of novel members of the allatostatin receptor family from insects. Biochem. Biophys. Res. Commun. 282, 904–9 (2001). 11352636 10.1006/bbrc.2001.4659

[b32] Yang J. . The I-TASSER Suite: Protein structure and function prediction. Nat. Methods 12, 7–8 (2015). 25549265 10.1038/nmeth.3213 PMC4428668

[b33] Trott O. & Olson A. J. Software News and Update AutoDock Vina : Improving the Speed and Accuracy of Docking with a New Scoring Function. Efficient Optimization. and Multithreading. J Comput Chem 31, 455–461 (2010). 19499576 10.1002/jcc.21334 PMC3041641

[b34] Humphrey W., Dalke A. & Schulten K. VMD - Visual Molecular Dynamics. J. Molec. Graph. 14, 33–38 (1996). 8744570 10.1016/0263-7855(96)00018-5

[b35] Phillips J. C. . Scalable molecular dynamics with NAMD. J. Comput. Chem. 26, 1781–1802 (2005). 16222654 10.1002/jcc.20289 PMC2486339

