# Supplementary material for: Ligand binding pocket of a novel Allatostatin receptor type C of stick insect, Carausius morosus
Source: Sci Rep. 2017 Jan 24;7:41266. doi: 10.1038/srep41266 (PMC5259779; doi:10.1038/srep41266)
Supplement: Supplementary Information [file srep41266-s1.pdf]

Ligand binding pocket of a novel Allatostatin receptor type C of stick insect, *Carausius morosus*

Burcin Duan Sahbaz[1], Osman Ugur Sezerman[2], Hamdi Torun[3], Necla Birgül Iyison[1]*

Supplementary Information

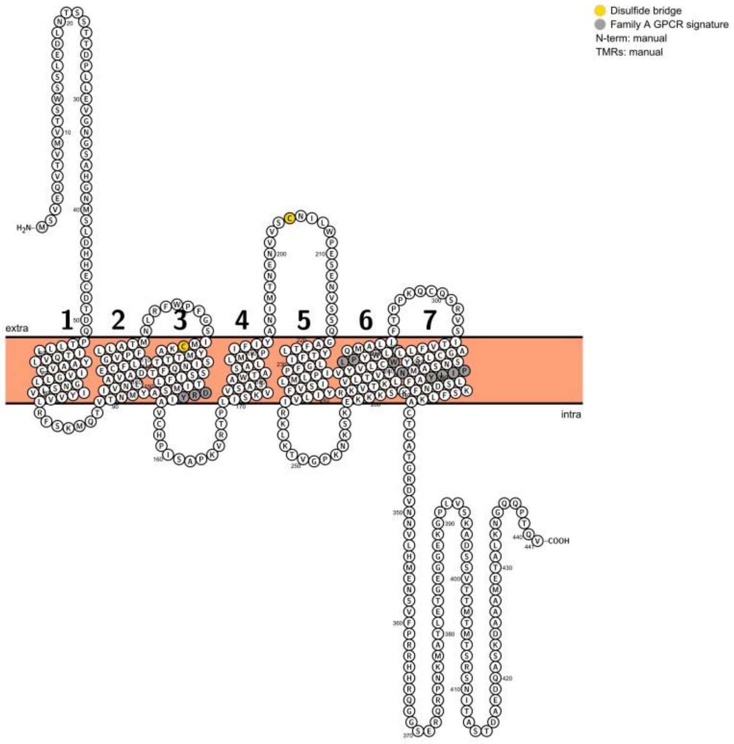

Supplementary Figure S1: The snake plot diagram of CamAlstR-C. The conserved Family A GPCR patterns were shown in grey and the cysteines that form a bridge were shown in yellow. The diagram was obtained from PROTTER web application).

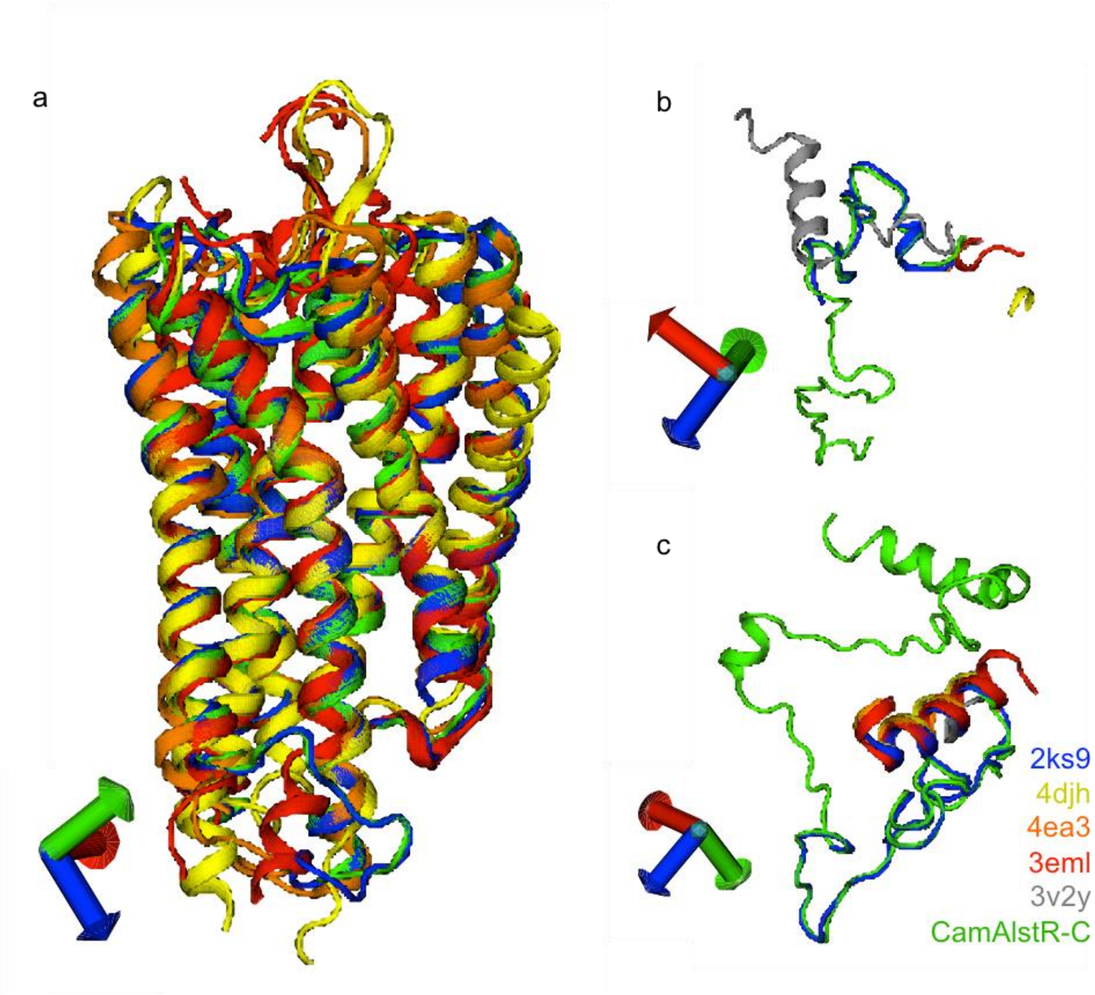

Supplementary Figure S2: The structural alignment of different templates. (a) Transmembrane, **(b)** N-terminal and **(c)** C-terminal regions of templates (2ks9, 4djh, 4ea3, 3eml and 3v2y) with the CamAlstR-C model.

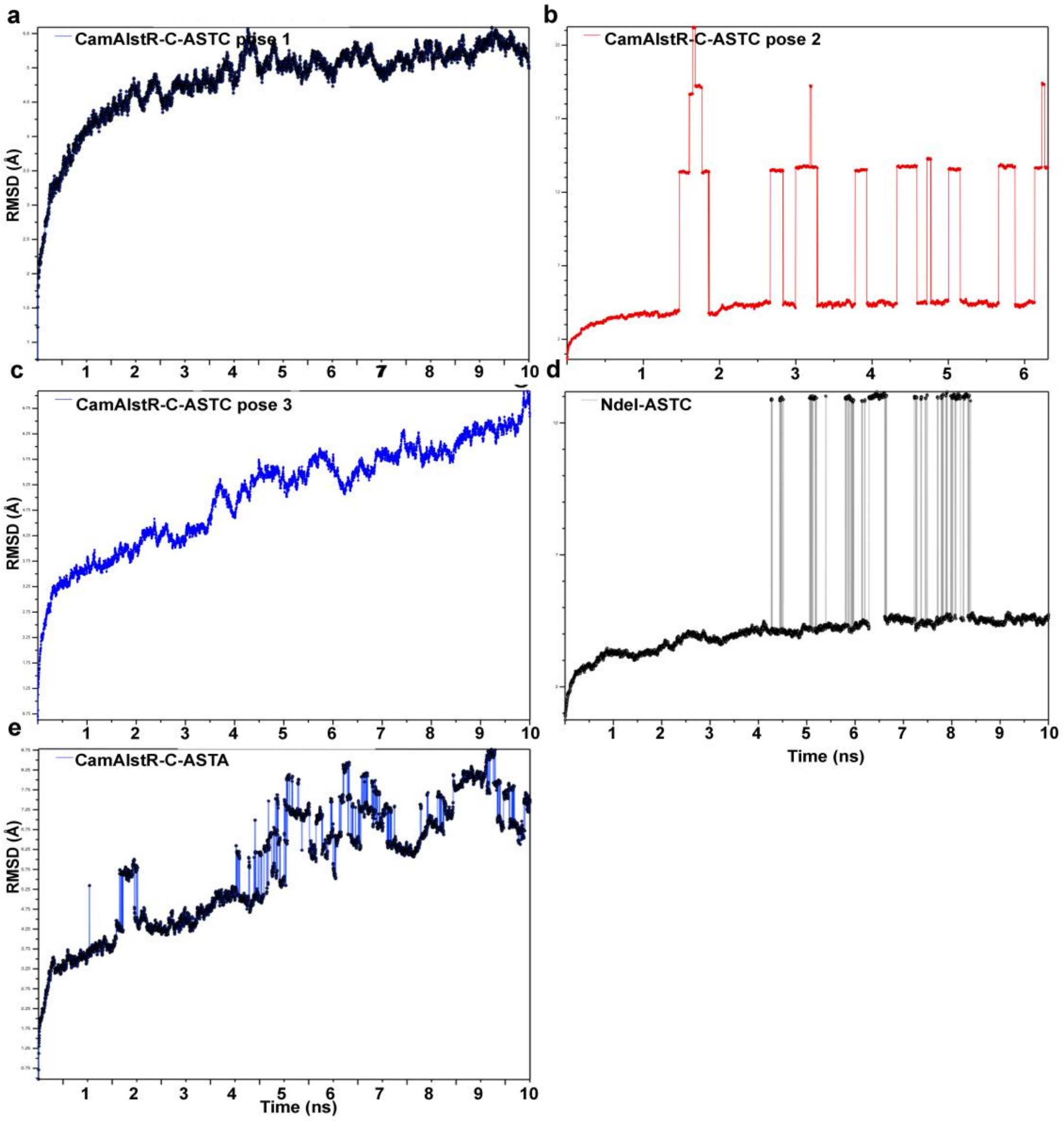

Supplementary Figure S3: Root Mean Square Deviations (RMSD) of receptor-ligand systems during 10 ns molecular dynamics simulations. **(a)** The simulations performed with wild type CamAlstR-C and AST-C peptide with the pose obtained from docking region I (pose 1), **(b)** from docking region II and **(c)** from docking region III were plotted. In addition, **(d)** the simulation performed with N-terminus deleted receptor (Ndel) and AST-C peptide and **(e)** the simulation performed with wild type CamAlstR-C and AST-A peptide were plotted.

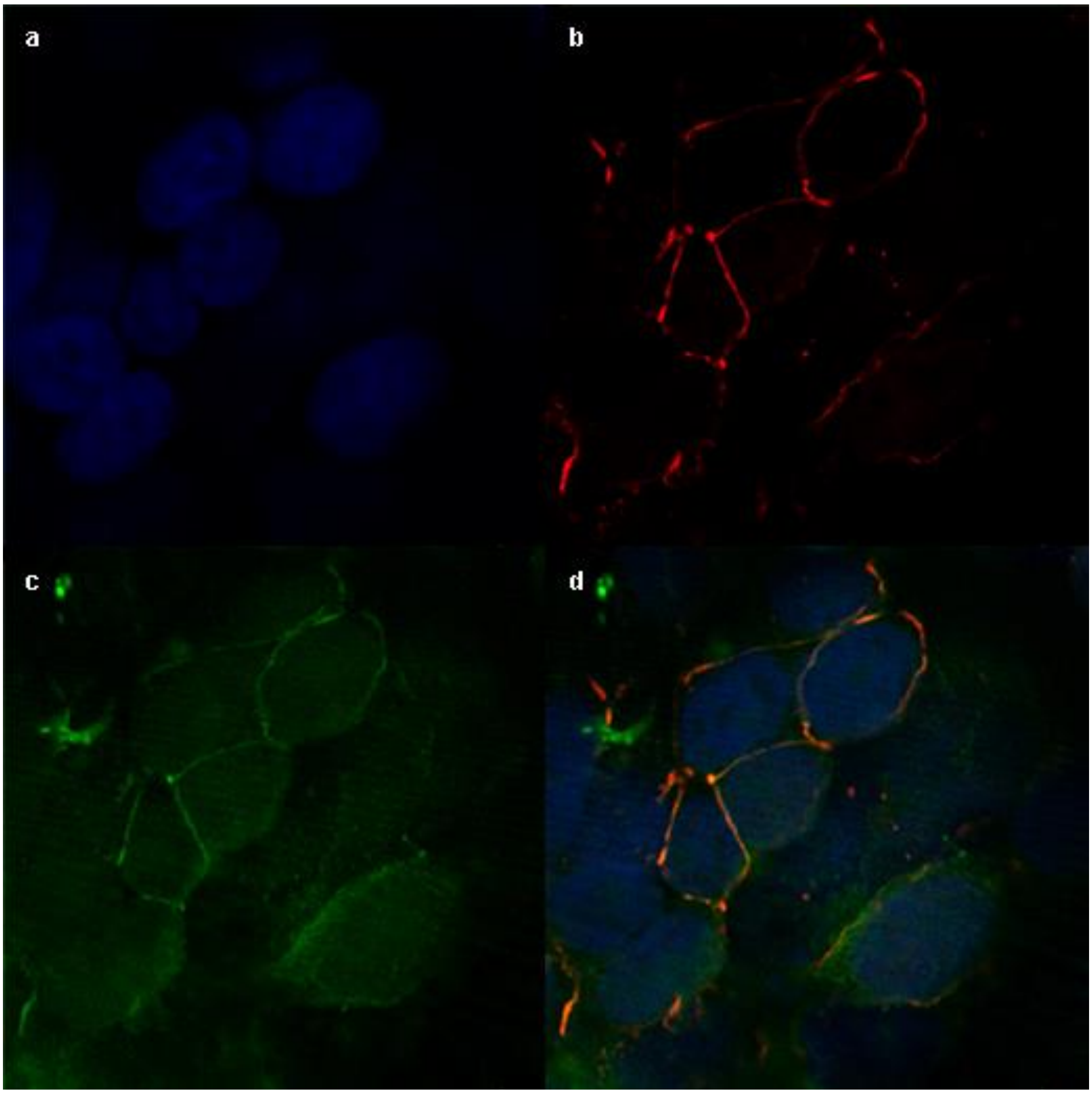

Supplementary Figure S4: Surface localization of CamAlstR-C.

Transiently transfected Huh7 cells were stained with **(a)** DAPI (blue) and immunostained with **(b)** anti-ZO1 (red) for the membrane. SYFP fused CamAlstR-C (green) expression **(c)** was colocalized with the membrane marker ZO1 proteins **(d)**.

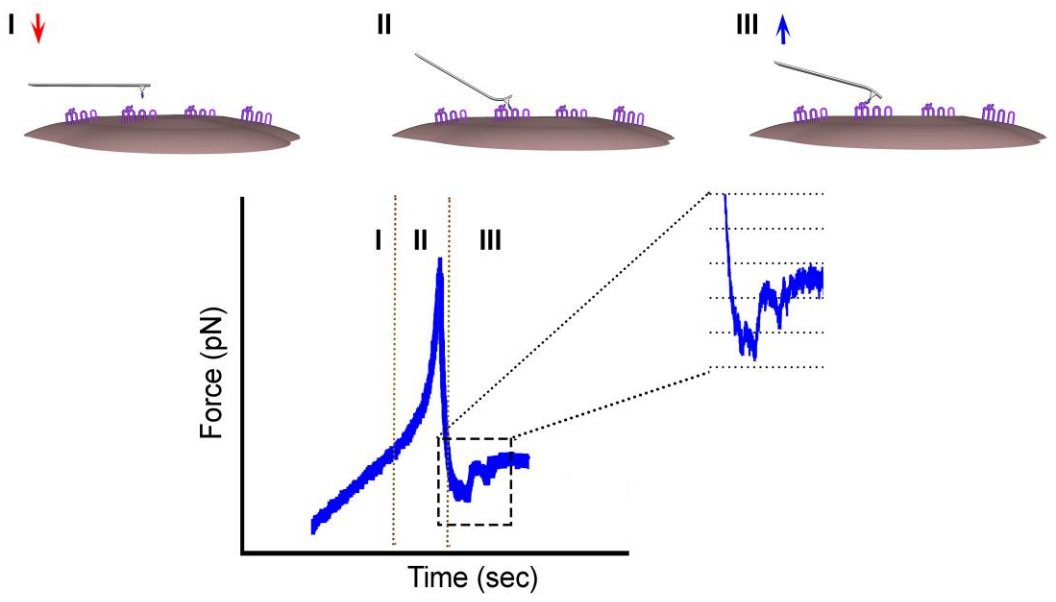

Supplementary Figure S5: Schematic overview of AFM set up and corresponding Force-Time curves. The cantilever with the peptide approaches to the cell surface (I). During the contact with the cell membrane the laser deflection shows a sharp increase and decrease (II). After equilibration point during retraction, unbinding events occur (III) and magnitudes of these events are evaluated as rupture forces.

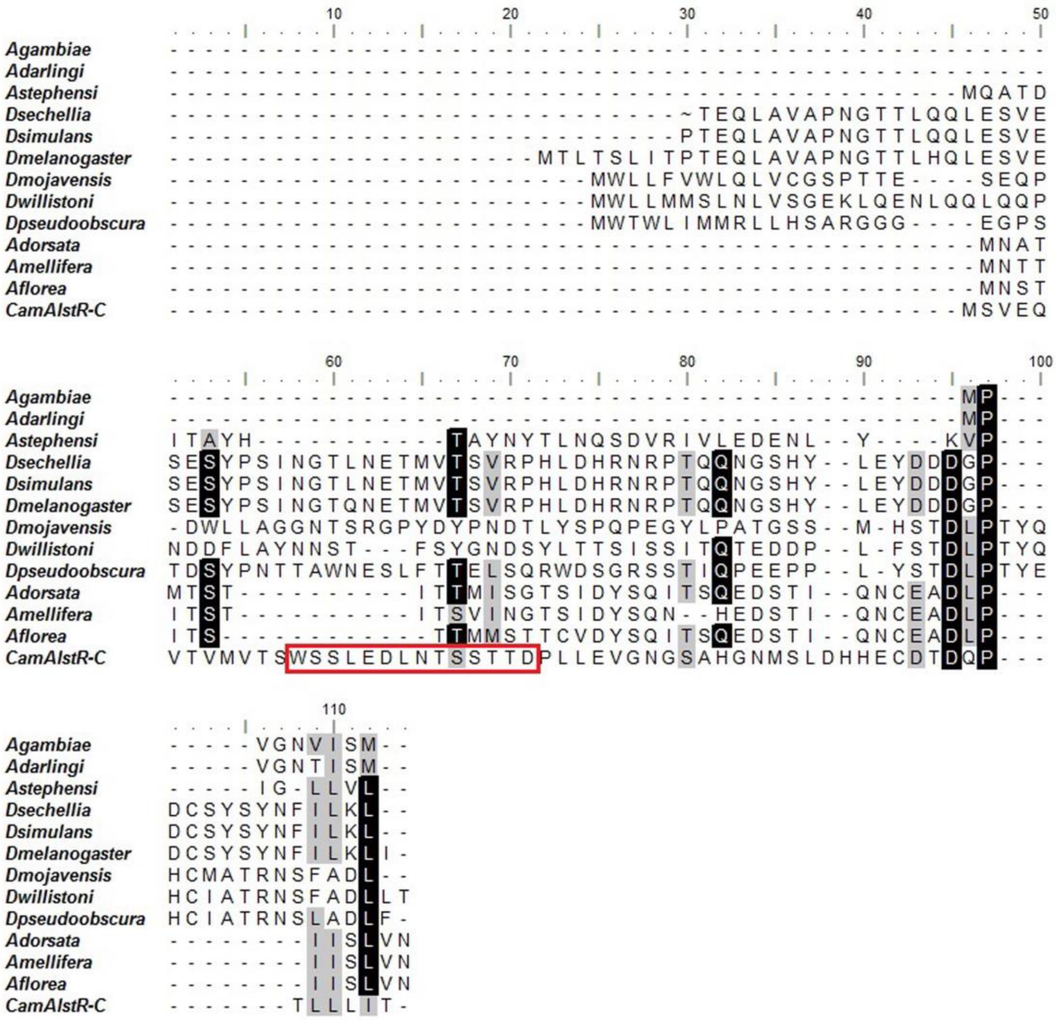

Supplementary Figure S6: Variability of N-terminal region between different genera. N-terminal regions of AlstR-C receptors from different species showed no overall conservation but exhibited partial conservation within a genus. The italic names correspond to the first letter of genus name followed by species name. Black and gray highlights show the identical and similar residues respectively. Red rectangle represents the interacting residues obtained from docking.

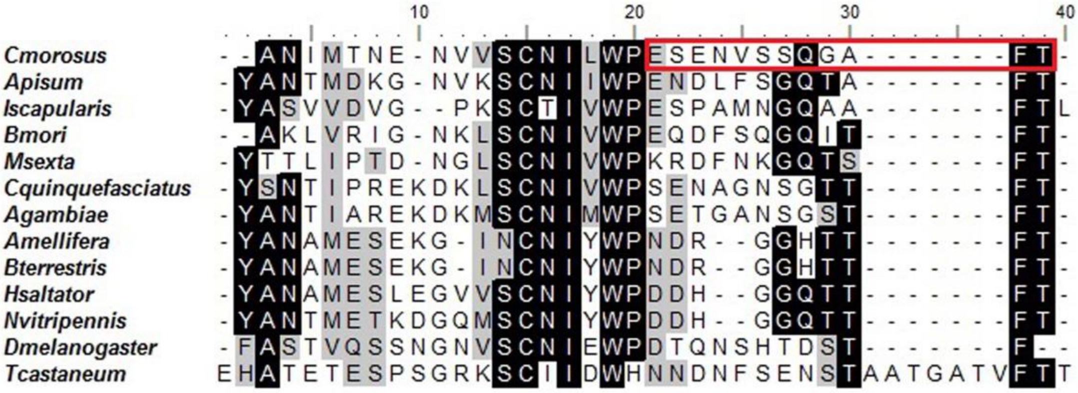

Supplementary Figure 7: Conservation on ECL2. ECL2 exhibits partial conservation within thirteen species. Red rectangle represents the interacting residues obtained from docking. Interacting residues are placed in a variable region residing right next to a highly conserved sequence motif (SCNIXWP).

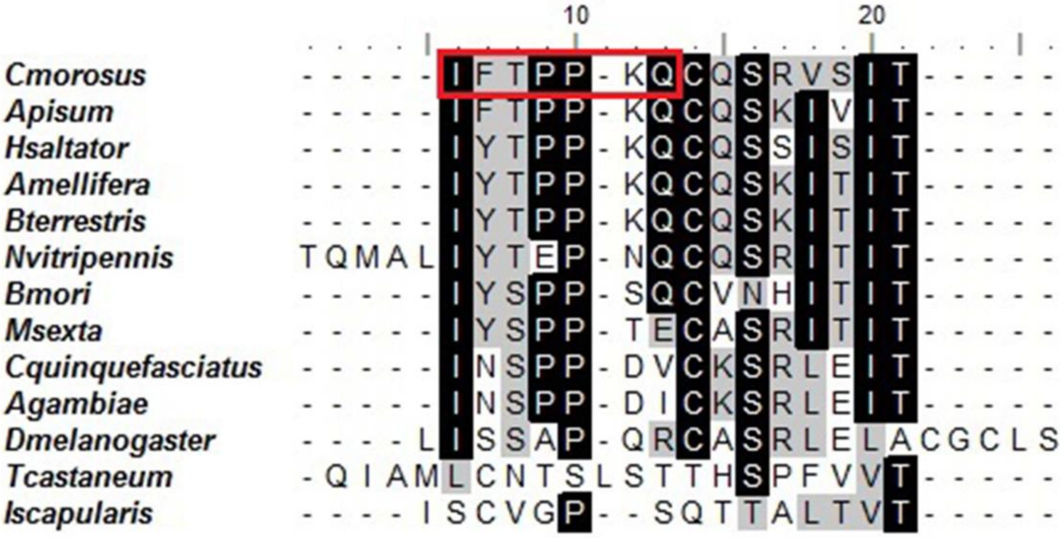

Supplementary Figure S8: Conservation on ECL3. The most conserved loop was ECL3. The interacting residues (red rectangle) were also very conserved within thirteen species.

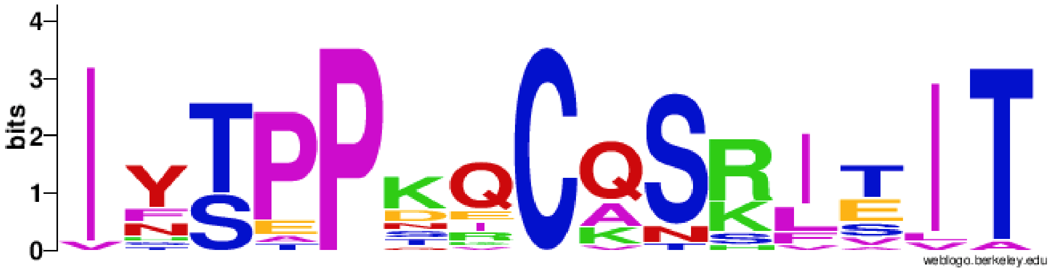

Supplementary Figure S9: Sequence logo on ECL3. The frequencies of amino acids on ECL3 were assessed in weblogo tool of Berkeley University. Within the interacting residues I292, T294, P295 and P296 were the most conserved ones.

Supplementary Table S1: Mutation codes and descriptions.

Abbreviation used in the text	Description of the mutation
WT	*Wild type*
Ndel	*Deletion of N-terminal 50 amino acids*
AFTPP	*I292A substitution*
AFTPA	*I292A and P296A substitutions*
AFAPA	*I292A, T294A and P296A substitutions*
AATPA	*I292A, F293A and P296A substitutions*
AFAAA	*I292A, T294A, P295A and P296A substitutions*
AAAAA	*I292A, F293A, T294A, P295A and P296A substitutions*

Supplementary Table S2: Bell's parameters for interaction of AST-C with different forms of CamAlstR-C.

**Forms of CamAlstR-C**	**K_{off} (s^{-1})**	**x_β (Å)**
WT (at low loading rates)	2,00E+10	0,828
WT (at high loading rates)	3,33E+09	0,138
Ndel	2,50E+10	1,040
AAAAA	5,00E+11	2,070
AFAAA	1,00E+11	4,140
AATPA	5,00E+10	2,070
AFAPA	1,11E+11	4,600
AFTPA	1,00E+11	4,140
AFTPP	5,00E+10	2,070

Supplementary Table S3: Primer sequences and codes used in the project. The nucleotides in Bold correspond to the mutated nucleotides.

Mutation code	Primer code	Sequence (5'-3')
WT	WT-ATG	AAAGCTTATCTAGAAAAATGTCTGTGGAACAAGTGACG
WT	WT-stop	TTTGAATTCTTGGATCCTCTACACCTGGGTCGGCTG
NdeI	NdeI-ATG	AAAAAGCTTATGGACACAGACCAGCCGACG
AFTPP	F1	ACGCAGATGGCGCTC**GC**CTTCACGCCGCCCAA
AFTPP	R1	TTGGGCGGCGTGAAGGCGAGCGCCATCTGCGT
AFTPA	F11	TCATCTTCACGCCG**G**CCAAGCAGTGCCAGT
AFTPA	R11	ACTGGCACTGCTTGGCCGGCGTGAAGATGA
AATPA	F111	AGATGGCGCTCGCC**GC**CACGCCGGCCAA
AATPA	R111	TTGGCCGGCGTGGCGGCGAGCGCCATCT
AFAPA	F112	ATGGCGCTCGCCTTC**GCC**CCGGCCAAGCAGT
AFAPA	R112	ACTGCTTGGCCGGGGCGAAGGCGAGCGCCAT
AFAAA	F1121	TCGCCTTCGCC**GCC**GCCAAGCAGT
AFAAA	R1121	ACTGCTTGGCGGCGGCGAAGGCGA
AAAAA	F11211	AGATGGCGCTCGCC**GC**CGCCGCCGCCAAGC
AAAAA	R11211	GCTTGGCGGCGGCGGCGGCGAGCGCCATCT
